# The relationship between shrunken pore syndrome and all-cause mortality in people with type 2 diabetes and normal renal function: the Fremantle Diabetes Study Phase II

**DOI:** 10.1007/s00125-025-06430-6

**Published:** 2025-04-21

**Authors:** David G. Bruce, Wendy A. Davis, S. A. Paul Chubb, Timothy M. E. Davis

**Affiliations:** Medical School, University of Western Australia, Fremantle, WA Australia

**Keywords:** Creatinine, Cystatin C, Estimated glomerular filtration rate, Mortality, Type 2 diabetes

## Abstract

**Aims/hypothesis:**

Estimated GFRs utilising creatinine- (eGFR_creat_) or cystatin C-based (eGFR_cyst_) equations can generate discrepant results that are associated with clinical outcomes. A low eGFR_cyst_/eGFR_creat_ ratio (<0.60), reflecting a pathological glomerular state termed shrunken pore syndrome (SPS), has been associated with excess mortality in some clinical situations including diabetes. The aim of the present study was to explore this association in a longitudinal observational study of type 2 diabetes with special reference to participants with normal renal function.

**Methods:**

Of 1481 Fremantle Diabetes Study Phase II participants with type 2 diabetes, aged ≥17 years, 1466 had eGFR_creat_ and eGFR_cyst_ assessed as part of the baseline assessment and were followed for 10 years or until death, whichever came first. Cox regression modelling was used to determine independent associates of death excluding eGFR; eGFR_cyst_/eGFR_creat_ ratio was then added to this model separately as a categorical or continuous variable. These analyses were also conducted in a subgroup (*n*=754) of participants with normal renal function (eGFR_creat_ ≥60 ml/min per 1.73 m^2^ and urinary albumin/creatinine ratio <3 mg/mmol) at baseline.

**Results:**

At entry, the participants had a mean age of 65.9 years, 51.8% were male, the median diabetes duration was 9.0 years and 10.4% had eGFR_cyst_/eGFR_creat_ ratio <0.60 (the definition of SPS). There were 384 deaths (26.2%) during follow-up. The eGFR_cyst_/eGFR_creat_ ratio was independently, significantly and negatively associated with death (adjusted HR [95% CI] 0.91 [0.85, 0.97] for an increase of 0.1, *p*=0.004). Of eGFR_cyst_/eGFR_creat_ ratio categories, only <0.60 added significantly to the most parsimonious Cox model of time to death (HR [95% CI] 1.56 [1.07, 2.29], *p*=0.021). In those with normal renal function, 123 (16.3%) died during follow-up. An eGFR_cyst_/eGFR_creat_ ratio <0.60, observed in 57 (7.6%), was also independently associated with mortality (HR [95% CI] 2.55 [1.34, 4.84], *p*=0.004).

**Conclusions/interpretation:**

A low eGFR_cyst_/eGFR_creat_ ratio is independently associated with mortality in type 2 diabetes, including in people without conventional markers of diabetic kidney disease. The presence of SPS may add clinical value to the risk assessment of people with type 2 diabetes regardless of renal status.

**Graphical Abstract:**

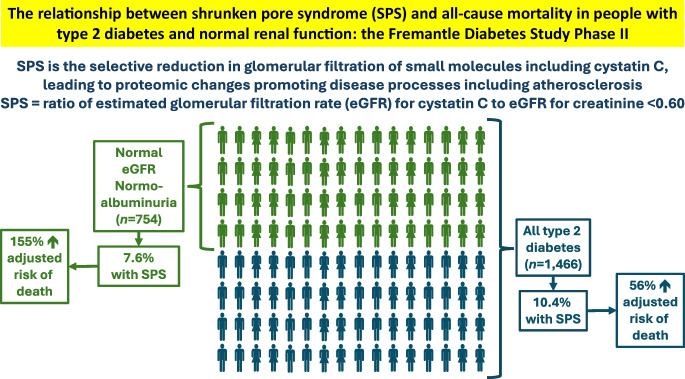


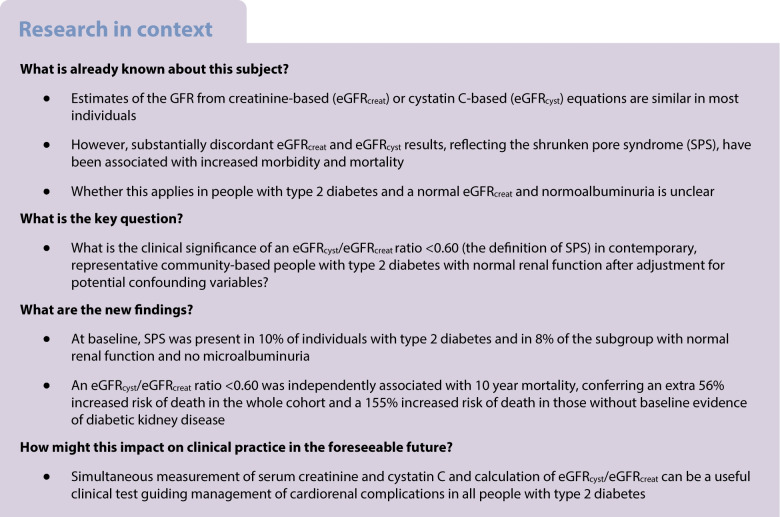


## Introduction

Estimates of the GFR (eGFR) are similar regardless of whether they are generated from creatinine-based (eGFR_creat_) or cystatin C-based (eGFR_cyst_) equations in most individuals. However, substantially discordant eGFR_creat_ and eGFR_cyst_ results have been found to be associated with increased morbidity and mortality [1]. The shrunken pore syndrome (SPS) has been proposed to explain this observation and a range of associated pathophysiological findings [2]. SPS has been suggested as one of several potential selective glomerular hypofiltration syndromes and may be one of the earliest markers of renal dysfunction [3]. The adverse clinical effects related to SPS may be explained by selective reduction in glomerular filtration of 5 kDa- to 30 kDa-sized molecules, including cystatin C, with a range of consequent proteomic changes promoting pathophysiological processes including atherosclerosis [3–5].

The presence of SPS has been defined by a ratio of eGFR_cyst_ to eGFR_creat_ of <0.60, although values above this may also have clinical value indicating a possible spectrum of effect [6]. SPS has been associated with increased mortality in several clinical situations including after coronary artery bypass surgery [7], in renal outpatient clinic attendees [4] and in the healthy elderly [8]. Analysis of prospective data from three large cohorts of people with diabetes of unspecified type (the Chinese Incident, Development, and Prognosis of Diabetic Kidney Disease study [INDEED], the National Health Nutrition Examination Survey [NHANES] in the USA, and the UK Biobank) has shown that an eGFR_cyst_/eGFR_creat_ ratio of <0.60 vs ≥0.60 is associated with an 89–158% increased risk of death after adjustment for a limited range of potentially confounding variables [9].

The presence of SPS may have clinical value in individuals with a normal eGFR and normoalbuminuria, and thus without conventional biochemical evidence of kidney disease [2, 4]. Whether this is applicable to diabetes is unknown. Diabetes deserves special consideration in this context since the accuracy of various equations for estimating GFR have been questioned in diabetes when compared with formal measurement of GFR [10]. In addition, plasma cystatin C concentrations are increased in obesity, potentially confounding eGFR_cyst_ measurement in diabetes given that it is a frequent comorbidity [11]. The clinical value and cost-effectiveness of measuring plasma cystatin C in addition to routine serum creatinine assays in a subgroup of people with diabetes who may be perceived as having a homogeneously relatively good prognosis needs to be established.

The general aim of the present study was to confirm whether the eGFR_cyst_/eGFR_creat_ ratio has clinical utility, as found in data from INDEED, NHANES and the UK Biobank [9], in well-characterised people with type 1 or type 2 diabetes in the longitudinal community-based Fremantle Diabetes Study Phase II (FDS2) after adjusting for a comprehensive set of potentially confounding variables. The specific aim was to assess whether a low ratio (<0.60) is independently associated with mortality among FDS2 participants who had normal conventional indices of renal function at baseline.

## Methods

### Participants and approvals

The FDS2 is a prospective study of representative individuals with diabetes living in the primary catchment area of Fremantle Hospital in the Australian state of Western Australia (WA) [12]. The FDS2 cohort comprises 1732 participants, including 1482 (85.6%) with a clinical diagnosis of type 2 diabetes after excluding those found to have MODY or latent autoimmune diabetes of adults and 139 with a diagnosis of type 1 diabetes [13]. Detailed information regarding recruitment and classification of diabetes has been published [12, 13]. The FDS2 protocol was approved by the Southern Metropolitan Area Health Service Human Research Ethics Committee. All participants gave informed consent before participation. The FDS2 was carried out in accordance with the principles of the Declaration of Helsinki.

### Clinical and biochemical data

All participants underwent a detailed assessment at study entry that included a comprehensive questionnaire, a clinical examination and collection of fasting blood and urine samples. Each participant was invited to return for biennial assessments, with additional biennial clinical questionnaires completed between face-to-face assessments. Racial/ethnic background was categorised based on self-selection, country/countries of birth and parents’/grandparents’ birth, and language(s) spoken at home as Anglo-Celt, Southern European, Other European, Asian, Aboriginal or mixed/other. BMI was determined together with a body shape index (ABSI), which represents a more reliable estimate of visceral adiposity [14]. Chronic complications were ascertained using standard criteria [12]. Standard-care biochemical testing of serum glucose, HbA_1c_, serum urea and electrolytes, serum lipids, serum uric acid, liver function, and urine albumin and creatinine were performed using standard automated methods in a single nationally accredited laboratory (PathWest Laboratories, Fremantle Hospital) using an Integra 800 analyser (Roche Diagnostics Australia, Castle Hill, NSW, Australia).

In addition to standard-care biochemical tests, serum N-terminal pro-brain natriuretic peptide (NT-proBNP) was measured on an Elecsys 2010 (Roche Diagnostics Australia) and C-reactive protein was measured on an Architect ci16200 analyser (Abbott Diagnostics Australia, North Ryde, NSW, Australia) using a high-sensitivity C-reactive protein (hsCRP) protocol with reagents supplied by Abbott Diagnostics. Serum cystatin C was measured by immunoturbidimetry (Multigent reagent kit; Abbott Diagnostics) on an Architect ci16200 analyser. The calibration of this assay is traceable to European Reference Material ERM-DA471/IFCC. Imprecision (expressed as the CV) was 4.7% at 0.56 mg/l and 1.2% at 4.1 mg/l. eGFRs were calculated from serum creatinine and from cystatin C by Chronic Kidney Diseases Epidemiology Collaboration (CKD-EPI) algorithms [15, 16]. An eGFR_cyst_/eGFR_creat_ ratio <0.60 was used to define SPS [2].

### Mortality ascertainment

The FDS2 cohort was followed from baseline (2008–2011) until end-December 2021 using ongoing FDS2 assessments and linkage through the Western Australian Data Linkage System (WADLS) [17], which includes the WA Registry for Births, Deaths and Marriages and the Hospital Morbidity Data Collection (HMDC). HMDC data supplemented those obtained through FDS2 assessments for pre-recruitment/prevalent disease between 1 January 1980 and study entry, and was used to extract the Charlson Comorbidity Index (CCI) [18] for the 5 years before study entry, excluding codes specific for diabetes and its complications. The primary outcome was all-cause mortality during the first 10 years of follow-up in all FDS2 participants with available data and in a subgroup without conventional markers of diabetic nephropathy at baseline (eGFR_creat_ ≥60 ml/min per 1.73 m^2^ and urinary albumin/creatinine ratio [uACR] <3.0 mg/mmol).

### Statistical analysis

Statistical analyses were performed using IBM SPSS Statistics for Windows (version 29; Armonk, NY, USA) or StataSE 15 (StataCorp LP, College Station, TX, USA). Data are presented as proportions, mean ± SD or geometric mean (SD range), or median [IQR] for variables that deviated from a normal distribution. For independent samples, two-way comparisons for proportions were by Fisher’s exact test, for normally distributed variables by Student’s *t* test and for non-normally distributed variables by Mann–Whitney *U* test. Cox regression identified independent determinants of all-cause mortality excluding eGFR. Backward stepwise entry was used with one variable manually removed at a time, the least significant first until all entered variables had *p*<0.05. The proportional hazards assumption was tested for using time-varying covariates and, when violated, adjusted for by adding the significant time-varying covariates. All clinically plausible variables with *p*≤0.20 in bivariable analyses were considered for entry (Model 1). Models 2 and 3 were then developed by adding eGFR_cyst_/eGFR_creat_ ratio categories and eGFR_cyst_/eGFR_creat_ ratio as a continuous variable, respectively, to the most parsimonious Model 1. This analytical approach was used for the whole FDS2 type 2 diabetes cohort and for the subgroup without evidence of diabetic kidney disease (DKD) at baseline.

## Results

### Participant characteristics

Of the 1481 eligible FDS2 participants with type 2 diabetes, 1468 (99.1%; mean ± SD age 65.9 ± 11.4 years, 51.8% male sex, median [IQR] diabetes duration 9.0 [3.0–15.9] years) had baseline blood samples that enabled calculation of eGFR_cyst_, eGFR_creat_ and eGFR_cyst_/eGFR_creat_. At baseline, 10.4% of the participants had an eGFR_cyst_/eGFR_creat_ ratio consistent with SPS (<0.60) and another 16.5% had an eGFR_cyst_/eGFR_creat_ between 0.60 and 0.69. Of the 133 eligible FDS2 participants with type 1 diabetes aged ≥17 years, 132 (99.2%; mean ± SD age 46.6 ± 16.5 years, 53.8% male sex, median [IQR] diabetes duration 20.0 [13.0–31.8] years) had baseline blood samples that enabled calculation of eGFR_cyst_, eGFR_creat_ and eGFR_cyst_/eGFR_creat_. At baseline, 10 (7.6%) had an eGFR_cyst_/eGFR_creat_ ratio <0.60.

### Mortality and its determinants

**Fig. 1:**
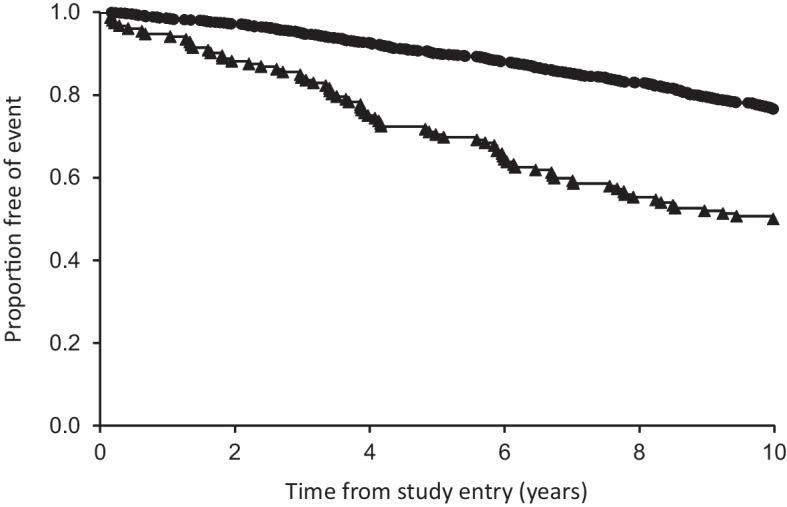
Kaplan–Meier curves of survival rate of participants in FDS2 with type 2 diabetes categorised by eGFR_cyst_/eGFR_creat_ ratio (circles, ≥0.60, *n*=1314; triangles, <0.60, *n*=152). The curves were significantly different (*p*<0.001 by logrank test)

Two participants with type 2 diabetes were lost to follow-up. The remaining 1466 were followed for 12,892 person-years (mean 8.8 ± 2.5 years) during which time 384 (26.2%) died. Baseline variables associated with all-cause death in bivariable analysis included older age, male sex, longer-duration diabetes, worse CVD risk factors and disease burden, taking more medications for CVD prevention, a greater number of comorbidities and diabetes-related chronic complications including worse indicators of kidney disease, specifically higher uACR, lower eGFR_creat_ and lower eGFR_cyst_/eGFR_creat_ ratios (see Table 1). A Kaplan–Meier plot of the two survival curves (eGFR_cyst_/eGFR_creat_ ≥0.60 and eGFR_cyst_/eGFR_creat_ <0.60) is shown in Fig. 1. The curves were significantly different (*p*<0.001 by logrank test).

**Table 1:** Baseline associates of all-cause mortality in participants with type 2 diabetes during the first 10 years of follow-up

Characteristic	Alive	Deceased	*p* value
*N* (%)	1082 (73.8)	384 (26.2)	
Age, years	63.4 ± 10.5	73.0 ± 10.9	<0.001
Male, %	50.0	57.0	0.020
Ethnic background, %			0.027
Anglo-Celt	52.3	57.3	
Southern European	12.4	13.0	
Other European	7.4	6.0	
Asian	5.2	2.3	
Aboriginal	6.2	8.6	
Mixed/other	16.5	12.8	
Not fluent in English, %	10.0	12.8	0.149
Educated beyond primary school, %	88.8	80.2	<0.001
Currently married/de facto relationship, %	67.1	50.0	<0.001
Alcohol consumption, standard drinks^a^/day	0.1 [0–1.2]	0.1 [0–1.5]	0.053
Smoking status, %			0.027
Never smoked	44.3	36.8	
Ex-smoker	45.9	50.5	
Current smoker	9.8	12.6	
Age at diabetes diagnosis, years	54.5 ± 11.4	59.2 ± 13.3	<0.001
Diabetes duration, years	7.0 [2.0–15.0]	14.0 [6.0–19.0]	<0.001
Diabetes treatment, %			<0.001
Diet	26.3	18.8	
Oral medications/non-insulin injectables	54.3	51.8	
Insulin alone	4.6	8.1	
Insulin ± oral medications/non-insulin injectables	14.8	21.4	
Fasting serum glucose, mmol/l	7.2 [6.2–8.7]	7.1 [6.0–8.9]	0.297
HbA_1c_, mmol/mol	51 [44–61]	52 [45–62]	0.041
HbA_1c_, %	6.8 [6.2–7.7]	6.9 [6.3–7.8]	0.041
BMI, kg/m^2^	31.6 ± 6.0	30.1 ± 6.0	<0.001
ABSI, m^11/6^ kg^−2/3^	0.081 ± 0.005	0.084 ± 0.005	<0.001
Heart rate, beats/min	69 ± 12	73 ± 14	<0.001
Systolic BP, mmHg	145 ± 21	149 ± 25	0.007
Diastolic BP, mmHg	81 ± 12	78 ± 13	0.003
Antihypertensive medications, %	72.4	81.7	<0.001
Total serum cholesterol, mmol/l	4.4 ± 1.1	4.3 ± 1.2	0.329
Serum HDL-cholesterol, mmol/l	1.23 ± 0.32	1.25 ± 0.38	0.437
Serum triglycerides, mmol/l	1.5 (0.9–2.5)	1.5 (0.9–2.5)	0.524
Lipid-modifying treatment, %	69.0	70.4	0.651
Aspirin use, %	35.1	45.9	<0.001
Clopidogrel use, %	7.5	12.6	0.004
Warfarin use, %	3.2	8.6	<0.001
Digoxin use, %	1.4	6.8	<0.001
eGFR (CKD-EPI) (ml/min per 1.73 m^2^)	84 ± 18	67 ± 25	<0.001
eGFR (CKD-EPI) categories, %			<0.001
≥90 ml/min per 1.73 m^2^	44.2	19.8	
60–89 ml/min per 1.73 m^2^	45.6	44.0	
45–59 ml/min per 1.73 m^2^	7.0	15.1	
30–44 ml/min per 1.73 m^2^	2.8	12.0	
<30 ml/min per 1.73 m^2^	0.5	9.1	
eGFR_cyst_/eGFR_creat_ ratio	0.84 ± 0.17	0.74 ± 0.18	<0.001
eGFR_cyst_/eGFR_creat_ ratio categories, %			<0.001
≥0.90	34.2	16.1	
0.80–0.89	22.8	16.4	
0.70–0.79	21.5	25.3	
0.60–0.69	14.4	22.4	
<0.60	0.8	19.8	
uACR, mg/mmol	2.8 (1.1–10.0)	5.3 (1.2–23.5)	<0.001
Serum uric acid, mmol/l	0.34 ± 0.08	0.36 ± 0.10	<0.001
Any retinopathy, %	34.2	46.5	<0.001
Distal symmetrical polyneuropathy, %	52.8	75.7	<0.001
Peripheral arterial disease, %	19.0	33.8	<0.001
Atrial fibrillation/flutter, (%)	2.6	10.6	<0.001
History of hospitalisation for/with atrial fibrillation/flutter, %	4.3	14.1	<0.001
CHD, %	24.8	43.2	<0.001
Cerebrovascular disease, %	5.8	16.7	<0.001
Prior hospitalisation with heart failure, %	3.0	15.1	<0.001
Plasma NT-proBNP, pmol/l	56 (17–192)	233 (48–1140)	<0.001
Plasma hsCRP, mg/l	2.4 (0.8–7.3)	2.7 (0.9–8.7)	0.058
CCI, %			<0.001
0	81.9	55.5	
1–2	13.8	26.8	
≥3	4.3	17.7	

Data are presented as percentages, mean±SD or geometric mean (SD range), or median [IQR]

^a^One standard drink=10 g pure alcohol

The results of Cox regression models for all-cause mortality in the participants with type 2 diabetes are presented in Table 2. Significant independent associates of death included age, male sex, obesity, smoking history, diabetes duration, peripheral arterial disease, comorbidities and several measures of CVD dysfunction. The addition of the eGFR_cyst_/eGFR_creat_ ratio (continuous variable, Model 3) was statistically significant, with a higher ratio being protective (HR 0.91 [95% CI 0.85, 0.97], *p*=0.004). The addition of eGFR_cyst_/eGFR_creat_ ratio category <0.60 was significantly and independently associated with an increased risk of death (Model 2; HR 1.56 [95% CI 1.07, 2.29], *p*=0.021).

**Table 2:** Independent associates of all-cause mortality in participants with type 2 diabetes during the first 10 years of follow-up

Variable	Model 1		Model 2		Model 3	
	HR (95% CI)	*p* value	HR (95% CI)	*p* value	HR (95% CI)	*p* value
Age (increase of 10 years)	1.71 (1.51, 1.94)	<0.001	1.65 (1.45, 1.88)	<0.001	1.63 (1.43, 1.86)	<0.001
Male	1.51 (1.20, 1.91)	0.001	1.58 (1.25, 2.00)	<0.001	1.55 (1.23, 1.97)	<0.001
Married/de facto relationship	0.72 (0.57, 0.90)	0.004	0.72 (0.58, 0.90)	0.004	0.73 (0.58, 0.91)	0.006
Current smoker	3.15 (1.89, 5.26)	<0.001	2.98 (1.78, 4.99)	<0.001	2.93 (1.75, 4.90)	<0.001
Diabetes duration (increase of 5 years)	1.09 (1.03, 1.16)	0.005	1.09 (1.02, 1.15)	0.006	1.09 (1.03, 1.16)	0.004
HbA_1c_ (increase of 11 mmol/mol or 1%)	1.10 (1.02, 1.19)	0.015	1.12 (1.03, 1.21)	0.007	1.11 (1.02, 1.20)	0.011
BMI (increase of 1 kg/m^2^)	0.97 (0.95, 0.99)	0.010	0.97 (0.94, 0.99)	0.001	0.97 (0.95, 0.99)	0.004
ABSI (increase of 0.001 m^11/6^ kg^−2/3^)	1.03 (1.01, 1.06)	0.007	1.03 (1.01, 1.05)	0.011	1.03 (1.01, 1.05)	0.010
Heart rate (increase of 10 beats/min)	1.22 (1.13, 1.32)	<0.001	1.22 (1.13, 1.32)	<0.001	1.22 (1.13, 1.32)	<0.001
Systolic BP (increase of 10 mmHg)	0.94 (0.90, 0.98)	0.004	0.94 (0.90, 0.99)	0.009	0.95 (0.91, 0.99)	0.013
Log_*e*_(NT-proBNP) (pmol/l)^a^	1.42 (1.31, 1.55)	<0.001	1.41 (1.29, 1.53)	<0.001	1.40 (1.29, 1.53)	<0.001
Log_*e*_(hsCRP) (mg/l)^a^	1.33 (1.11, 1.59)	0.002	1.31 (1.09, 1.56)	0.004	1.30 (1.08, 1.56)	0.005
Peripheral arterial disease	1.30 (1.03, 1.63)	0.025	1.29 (1.03, 1.63)	0.027	1.29 (1.03, 1.62)	0.028
CCI=1 or 2	1.35 (1.05, 1.75)	0.020	1.35 (1.04, 1.74)	0.022	1.35 (1.05, 1.75)	0.020
CCI ≥3	1.66 (1.22, 2.27)	0.001	1.71 (1.25, 2.34)	0.001	1.66 (1.22, 2.26)	0.001
eGFR_cyst_/eGFR_creat_ ratio:						
≥0.90			1.00			
0.80–0.89			0.84 (0.59, 1.20)	0.338		
0.70–0.79			1.36 (0.98, 1.88)	0.070		
0.60–0.69			1.22 (0.86, 1.73)	0.270		
<0.60			1.56 (1.07, 2.29)	0.021		
eGFR_cyst_/eGFR_creat_ ratio (increase of 0.1)					0.91 (0.85, 0.97)	0.004
Time-varying variables^b^						
Current smoker	0.60 (0.44, 0.83)	0.002	0.60 (0.44, 0.82)	0.001	0.60 (0.44, 0.82)	0.002
Log_*e*_(hsCRP) (mg/l)^a^	0.89 (0.80, 0.99)	0.032	0.89 (0.81, 0.99)	0.037	0.90 (0.81, 0.99)	0.038
Data missing for *n* (%) participants	24 (1.6%)		24 (1.6%)		24 (1.6%)	

The results are presented with HRs and 95% CIs as follows: (1) the most parsimonious model excluding eGFR (Model 1); then (2) entering eGFR_cyst_/eGFR_creat_ ratio categories (Model 2); and then (3) entering eGFR_cyst_/eGFR_creat_ ratio as a continuous variable (Model 3)

^a^An increase of 1 in log_*e*_(x) equates to an increase of 2.72 in x

^b^Time-varying variables interacted with log_*e*_(_t)

**Fig. 2:**
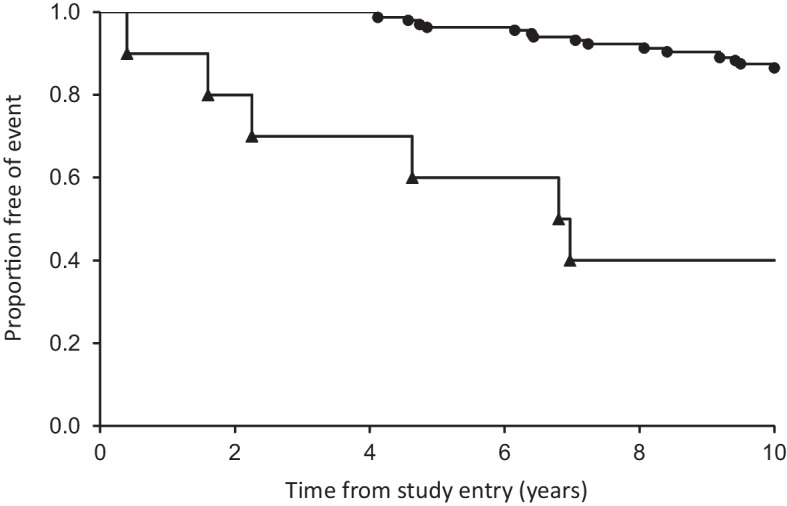
Kaplan–Meier curves of survival rate of participants in the FDS2 with type 1 diabetes categorised by eGFR_cyst_/eGFR_creat_ ratio (circles, ≥0.60, *n*=122; triangles, <0.60, *n*=10). The curves were significantly different (*p*<0.001 by logrank test)

In the subgroup with type 1 diabetes, there were 22 deaths (16.7%) during a mean ± SD 9.4 ± 1.8 years of follow-up. In Kaplan–Meier analysis, eGFR_cyst_/eGFR_creat_ <0.60 was significantly associated with death (logrank test, *p*<0.001; see Fig. 2). Participants with an eGFR_cyst_/eGFR_creat_ ratio <0.60 were significantly older than those without (67.9 ± 16.5 vs 44.9 ± 15.3 years, *p*<0.001). After adjustment for age in Cox regression analysis, the association between eGFR_cyst_/eGFR_creat_ <0.60 and death was no longer significant (*p*=0.989).

### Subgroup with normal renal function

The subgroup of 754 participants with type 2 diabetes and normal renal parameters at baseline were aged 62.9 ± 10.5 years at entry, 51.9% were male and their median [IQR] diabetes duration was 6.0 [1.6–14.0] years. Of these, 57 (7.6%) had eGFR_cyst_/eGFR_creat_ ratio <0.60 and another 107 (14.2%) had ratios between 0.60 and 0.69. During follow-up, 123 (16.3%) died. Table 3 summarises bivariable associations with mortality. Those who died were older, developed diabetes at an older age, had longer diabetes duration, more comorbidity, greater CVD burden, more complications, lower eGFR_creat_, higher uACR and were more likely to have an eGFR_cyst_/eGFR_creat_ ratio <0.60. The addition of eGFR_cyst_/eGFR_creat_ ratio as a continuous variable (Model 3) was statistically significant, with a higher ratio being protective (HR 0.84 [95% CI 0.75, 0.94], *p*=0.002; Table 4). The addition of eGFR_cyst_/eGFR_creat_ categories in Model 2 revealed a substantial independent increased risk of death for a ratio <0.60 (HR 2.55 [95% CI 1.34, 4.84], *p*=0.004).

**Table 3:** Baseline associates of all-cause mortality in participants with type 2 diabetes during the first 10 years of follow-up in the subgroup with eGFR_creat_ ≥60 ml/min per 1.73 m^2^ and normoalbuminuria

Characteristic	Alive	Deceased	*p* value
*N* (%)	631 (83.7)	123 (16.3)	
Age, years	61.8 ± 10.1	68.4 ± 10.9	<0.001
Male, %	50.4	59.3	0.076
Ethnic background, %			0.069
Anglo-Celt	54.5	58.5	
Southern European	9.8	14.6	
Other European	7.9	8.1	
Asian	5.2	1.6	
Aboriginal	3.6	5.7	
Mixed/other	18.9	11.4	
Not fluent in English, %	8.2	13.0	0.119
Educated beyond primary school, %	91.3	86.6	0.122
Currently married/de facto relationship, %	70.2	54.5	0.001
Alcohol consumption (standard drinks^a^/day)	0.3 [0–1.2]	0.1 [0–1.5]	0.680
Smoking status, %			0.027
Never smoked	45.3	35.2	
Ex-smoker	45.9	49.2	
Current smoker	8.7	15.6	
Age at diabetes diagnosis, years	54.0 ± 11.0	56.9 ± 13.2	0.028
Diabetes duration, years	5.0 [1.3–13.0]	12.0 [3.0–17.1]	<0.001
Diabetes treatment, %			0.632
Diet	28.7	28.5	
Oral medications/non-insulin injectables	55.3	51.2	
Insulin alone	3.6	4.9	
Insulin ± oral medications/non-insulin injectables	12.4	15.4	
Fasting serum glucose, mmol/l	7.1 [6.1–8.4]	6.9 [6.0–8.4]	0.652
HbA_1c_, mmol/mol	50 [43–57]	51 [44–58]	0.322
HbA_1c_, %	6.7 [6.1–7.4]	6.8 [6.2–7.5]	0.322
BMI, kg/m^2^	31.5 ± 5.8	30.1 ± 5.2	0.011
Central adiposity, % by waist circumference	69.5	65.6	0.394
ABSI, m^11/6^ kg^−2/3^	0.080 ± 0.005	0.083 ± 0.006	<0.001
Heart rate, beats/min	68 ± 11	73 ± 14	<0.001
Systolic BP, mmHg	142 ± 19	143 ± 20	0.387
Diastolic BP, mmHg	81 ± 11	78 ± 11	0.035
Antihypertensive medications, %	65.1	69.7	0.350
Total serum cholesterol, mmol/l	4.4 ± 1.0	4.3 ± 1.1	0.839
Serum HDL-cholesterol, mmol/l	1.23 ± 0.31	1.29 ± 0.40	0.122
Serum triglycerides, mmol/l	1.5 (0.9–2.4)	1.4 (0.8–2.2)	0.109
Lipid-modifying treatment, %	66.9	64.8	0.675
Aspirin use, %	31.9	40.2	0.093
Clopidogrel use, %	6.5	9.8	0.180
Warfarin use, %	1.6	4.1	0.079
Digoxin use, %	0.8	4.1	0.014
eGFR (CKD-EPI), ml/min per 1.73 m^2^	88 ± 13	85 ± 14	0.005
eGFR (CKD-EPI) categories, %			0.002
≥90 ml/min per 1.73 m^2^	49.8	34.1	
60–89 ml/min per 1.73 m^2^	50.2	65.9	
eGFR_cyst_/eGFR_creat_ ratio	0.84 ± 0.16	0.75 ± 0.17	<0.001
eGFR_cyst_/eGFR_creat_ ratio categories, %			<0.001
≥0.90	34.7	16.3	
0.80–0.89	25.5	22.0	
0.70–0.79	21.1	24.4	
0.60–0.69	13.0	20.3	
<0.60	5.7	17.1	
uACR, mg/mmol	1.3 (0.8–2.1)	1.5 (1.0–2.4)	0.002
Uric acid, mmol/l	0.33 ± 0.08	0.32 ± 0.08	0.312
Any retinopathy, %	29.5	39.7	0.032
Distal symmetrical polyneuropathy, %	51.0	68.9	<0.001
Peripheral arterial disease, %	16.3	24.6	0.037
Atrial fibrillation/flutter, %	1.3	6.7	0.001
History of hospitalisation for/with atrial fibrillation/flutter, %	1.7	6.5	0.006
CHD, %	21.1	34.1	0.002
Cerebrovascular disease, %	3.8	7.3	0.092
Prior hospitalisation for/with heart failure, %	2.1	8.1	0.002
Plasma NT-proBNP, pmol/l	43 (14–129)	103 (25–428)	<0.001
Plasma hsCRP, mg/l	2.2 (0.7–6.6)	2.4 (0.8–7.2)	0.426
CCI, %			<0.001
0	85.1	67.5	
1–2	11.6	20.3	
≥3	3.3	12.2	

Data are presented as percentages, mean±SD, geometric mean (SD range) or median [IQR]

^a^One standard drink=10 g pure alcohol

**Table 4:** Independent associates of all-cause mortality in participants with type 2 diabetes during the first 10 years of follow-up in the subgroup of participants with eGFR_creat_ ≥60 ml/min per 1.73 m^2^ and normoalbuminuria

Variable	Model 1		Model 2		Model 3	
	HR (95% CI)	*p* value	HR (95% CI)	*p* value	HR (95% CI)	*p* value
Age (increase of 10 years)	1.39 (1.12, 1.73)	0.002	1.37 (1.11, 1.70)	0.004	1.36 (1.10, 1.67)	0.004
Male sex	1.76 (1.18, 2.61)	0.005	1.91 (1.28, 2.85)	0.002	1.91 (1.28, 2.84)	0.001
Currently married/de facto relationship	0.67 (0.46, 0.98)	0.038	0.66 (0.45, 0.97)	0.034	0.65 (0.45, 0.95)	0.027
ABSI (increase of 0.001 m^11/6^ kg^−2/3^)	1.05 (1.01, 1.09)	0.006	1.04 (1.01, 1.08)	0.019	1.04 (1.01, 1.08)	0.017
Heart rate (increase of 10 beats/min)	1.38 (1.20, 1.59)	<0.001	1.38 (1.20, 1.59)	<0.001	1.37 (1.19, 1.58)	<0.001
Diastolic BP (increase of 5 mmHg)	0.90 (0.82, 0.98)	0.014	0.90 (0.83, 0.99)	0.021	0.91 (0.83, 0.99)	0.026
Log_*e*_(plasma NT-proBNP) (pmol/l)^a^	1.58 (1.34, 1.81)	<0.001	1.55 (1.31, 1.84)	<0.001	1.55 (1.31, 1.83)	<0.001
eGFR_cyst_/eGFR_creat_ ratio:						
≥0.90			1.00			
0.80–0.89			1.32 (0.74, 2.37)	0.349		
0.70–0.79			1.70 (0.96, 3.02)	0.070		
0.60–0.69			1.51 (0.82, 2.77)	0.183		
<0.60			2.55 (1.34, 4.84)	0.004		
eGFR_cyst_/eGFR_creat_ ratio (increase of 0.1)					0.84 (0.75, 0.94)	0.002
Data missing for *n* (%) participants	7 (0.9)		7 (0.9)		7 (0.9)	

The results are presented with HRs and 95% CIs as follows: (1) the most parsimonious model excluding eGFR (Model 1); then (2) entering eGFR_cyst_/eGFR_creat_ ratio categories (Model 2); and then (3) entering eGFR_cyst_/eGFR_creat_ ratio as a continuous variable (Model 3)

^a^An increase of 1 in log_*e*_(x) equates to an increase of 2.72 in x

Because of the low numbers of FDS2 participants with type 1 diabetes and normal renal function variables at baseline (*n*=92 or 69.2%) of whom only three (3.3%) had SPS, a separate analysis of the relationship between eGFR_cyst_/eGFR_creat_ ratio and mortality was not performed.

## Discussion

The present study provides evidence that supports the clinical value of assessing criteria for SPS in people with type 2 diabetes regardless of their baseline renal function. Specifically, we found that an eGFR_cyst_/eGFR_creat_ ratio of <0.60, the current criterion defining the presence of SPS [6], was significantly and independently associated with 10 year mortality in representative, community-based participants with type 2 diabetes after adjustment for a wide range of potentially confounding variables. The impact was large, conferring a 56% increased risk of death compared with those with an eGFR_cyst_/eGFR_creat_ ratio ≥0.60. However, the effect was even greater in the participants who had normoalbuminuria and a normal eGFR at baseline, with a 155% increased risk of death when the eGFR_cyst_/eGFR_creat_ ratio was <0.60. SPS was not uncommon in the FDS2 cohort, affecting around 10% of study participants overall at baseline including 7.6% of those without conventional biochemical markers of DKD. These findings suggest that assessing eGFR using both creatinine- and cystatin C-based equations to determine the ratio of the two is likely to add value to the clinical assessment of all individuals with type 2 diabetes and assist with identification of people who may benefit from intensification of measures that prevent the development of CVD and DKD, and perhaps other complications associated with mortality.

Our results for type 2 diabetes are consistent with those published recently from three other large diabetes cohorts [9], although with an increased risk of death (56%) that was lower than in those of the other studies (89–158%). This lower relative mortality likely reflects, at least in part, the larger range of variables available for inclusion in adjusted models in the present analyses. Another difference was the baseline prevalence of SPS, which was 10% in the representative, community-based FDS2 participants with type 2 diabetes and thus greater than that in the other three cohorts (2.0–4.0%) [9] which may have involved relatively healthy people with diabetes [19]. In addition, the three other diabetes-specific studies would have included variable numbers of people with type 1 and other diabetes types [9] that could account for around one in every seven cases [13] and which may have a different impact on the relationship between eGFR_cyst_/eGFR_creat_ ratio and death compared with type 2 diabetes. In the present analyses of available data from the limited number of participants with type 1 diabetes, the increased mortality associated with an eGFR_cyst_/eGFR_creat_ ratio <0.60 was explained by age, but larger scale studies would be needed to explore this association in more detail. Nevertheless, it is possible that factors such as suboptimal glycaemic control and microvascular disease that underlie the relatively high mortality rate in type 1 vs type 2 diabetes [20, 21] could diminish a contribution by SPS.

An eGFR_cyst_/eGFR_creat_ ratio <0.60 was independently associated with subsequent mortality in people with type 2 diabetes and normal indices of renal function (uACR and eGFR) in the present study. This relationship has also been found in older people from the general population [8] and general hospital outpatient clinic attendees [22] but has not been assessed previously in the context of type 2 diabetes. The greater increased risk of death associated with an eGFR_cyst_/eGFR_creat_ ratio <0.60 in this subgroup (155%) compared with the type 2 diabetes cohort as a whole (56%) might be explained by the greater contribution of CVD and its risk factors to mortality in the whole cohort (as in the comparison of variables in Table 2 vs Table 4).

The term SPS is based on the pore model of glomerular disease, which proposes that a decrease in the diameter of a fraction of the pores of the glomerular membrane impairs the filtration of larger molecules including cystatin C to a greater extent than smaller molecules such as water or creatinine [2]. A recent study in people with DKD found that thickening of the glomerular basement membrane was inversely correlated with the eGFR_cyst_/eGFR_creat_ ratio, consistent with the SPS hypothesis [23]. In addition, a study carried out in Japanese adults with diabetes found that a greater eGFR_cyst_ than eGFR_creat_ category was associated with mortality, although the authors did not interpret their results in terms of eGFR ratios or SPS [24]. It seems likely that associations with mortality are explained in part by proteomic changes associated with SPS [3], including an increased propensity for atherogenesis that is reflected by selective reduction in filtration of 5–30 kDa molecules such as cystatin C and others, some of which may have causative pathological associations [4].

Our results have potential clinical implications. Current Kidney Disease Improving Global Outcomes guidelines endorse the use of eGFR_cyst_ for confirmatory testing in specific circumstances when the eGFR_creat_ is likely to be less accurate, such as with low muscle mass [25, 26]. However, in both the general population and people with diabetes, the value of assessing eGFR using cystatin C continues to be debated given that eGFR_cyst_ correlates only fairly with measured GFR and is not substantially superior to eGFR_creat_ in this regard [10, 27]. In addition, other international guidelines do not support the use of cystatin C measurement in chronic kidney disease management [28]. Another major consideration is the greater cost of cystatin C compared with creatinine measurement as the reagent cost for a cystatin C test is approximately AUD $6 (USD $4) compared with AUD $1 (USD $0.70) for a creatinine measurement by modern enzymatic methods.

While eGFR_cyst_ appears superior to eGFR_creat_ in determining risk of all-cause and cardiovascular mortality [1], our data suggest that measuring both simultaneously may have additional clinical value in type 2 diabetes. Such dual measurement may be particularly valuable early in the course of diabetes before the appearance of established predictors of DKD, as was seen in one in 13 of the FDS2 participants who had no microalbuminuria or reduction in eGFR at baseline. Although an analysis of the relationship between the eGFR_cyst_/eGFR_creat_ ratio and specific causes of death was beyond the scope of the present study and would be constrained by a lack of statistical power, reported associations between SPS and adverse cardiorenal outcomes [3–5] suggest that these individuals would benefit from more aggressive CVD risk reduction and/or measures aimed at reducing the risk of future DKD. There is the need for further studies to explore this hypothesis.

The present study has limitations. First, observational studies can be affected by bias related to study recruitment and retention. Another methodological limitation relates to the Cox modelling where, to avoid collinearity, the mortality hazards were assessed without including eGFR_creat_, a known powerful risk factor, in the parsimonious model before entering the eGFR_cyst_/eGFR_creat_ ratio. The use of stepwise variable selection also has limitations, with the potential for biased coefficients and *p* values, and inflated model fit statistics. The study strengths include the representative nature of the FDS2 cohort, the comprehensive nature of the baseline assessment and the long-running validated WADLS, which systematically collects accurate data on all hospitalisations and deaths in the state of WA.

In conclusion, notwithstanding issues relating to assay cost, the present data support simultaneous measurement of serum creatinine and cystatin C and calculation of a simple ratio (eGFR_cyst_/eGFR_creat_) in all people with type 2 diabetes as a useful clinical test for the development of cardiorenal complications associated with mortality. This includes individuals with previously normal renal function by conventional indices (eGFR and uACR).

## Data Availability

Restrictions apply to the availability of data generated or analysed during this study to preserve patient confidentiality or because they were used under license. The corresponding author will on request detail the restrictions and any conditions under which access to some data may be provided.

## Abbreviations

ABSI: A body shape index
CCI: Charlson Comorbidity Index
CKD-EPI: Chronic Kidney Diseases Epidemiology Collaboration
DKD: Diabetic kidney disease
eGFR: Estimated GFR
FDS2: Fremantle Diabetes Study Phase II
HMDC: Hospital Morbidity Data Collection
hsCRP: High-sensitivity C-reactive protein
INDEED: Chinese Incident, Development, and Prognosis of Diabetic Kidney Disease study
NHANES: National Health Nutrition Examination Survey
NT-proBNP: N-terminal pro-brain natriuretic peptide
SPS: Shrunken pore syndrome
uACR: Urinary albumin/creatinine ratio
WA: Western Australia
WADLS: Western Australian Data Linkage System

## References

[1] Shlipak MG, Matsushita K, Arnlov J et al (2013) Cystatin C versus creatinine in determining risk based on kidney function. N Engl J Med 369(10):932–943. 10.1056/NEJMoa1214234 24004120 10.1056/NEJMoa1214234 PMC3993094

[2] Grubb A (2020) Shrunken pore syndrome - a common kidney disorder with high mortality. Diagnosis, prevalence, pathophysiology and treatment options. Clin Biochem 83:12–20. 10.1016/j.clinbiochem.2020.06.002 32544475 10.1016/j.clinbiochem.2020.06.002

[3] Malmgren L, Oberg C, den Bakker E et al (2023) The complexity of kidney disease and diagnosing it - cystatin C, selective glomerular hypofiltration syndromes and proteome regulation. J Intern Med 293(3):293–308. 10.1111/joim.13589 36385445 10.1111/joim.13589 PMC10107454

[4] Almen MS, Bjork J, Nyman U et al (2019) Shrunken pore syndrome is associated with increased levels of atherosclerosis-promoting proteins. Kidney Int Rep 4(1):67–79. 10.1016/j.ekir.2018.09.002 30596170 10.1016/j.ekir.2018.09.002 PMC6308389

[5] Xhakollari L, Jujic A, Molvin J et al (2021) Proteins linked to atherosclerosis and cell proliferation are associated with the shrunken pore syndrome in heart failure patients: shrunken pore syndrome and proteomic associations. Proteomics Clin Appl 15(4):e2000089. 10.1002/prca.202000089 33682349 10.1002/prca.202000089

[6] Grubb A, Lindstrom V, Jonsson M et al (2015) Reduction in glomerular pore size is not restricted to pregnant women. Evidence for a new syndrome: “shrunken pore syndrome.” Scand J Clin Lab Invest 75(4):333–340. 10.3109/00365513.2015.1025427 25919022 10.3109/00365513.2015.1025427 PMC4487590

[7] Dardashti A, Nozohoor S, Grubb A, Bjursten H (2016) Shrunken pore syndrome is associated with a sharp rise in mortality in patients undergoing elective coronary artery bypass grafting. Scand J Clin Lab Invest 76(1):74–81. 10.3109/00365513.2015.1099724 26647957 10.3109/00365513.2015.1099724 PMC4720044

[8] Purde MT, Nock S, Risch L et al (2016) Ratio of cystatin C and creatinine-based estimates of the glomerular filtration rate predicts mortality in healthy seniors independent of kidney function. Scand J Clin Lab Invest 76(4):341–343. 10.3109/00365513.2016.1149882 26981764 10.3109/00365513.2016.1149882

[9] He D, Gao B, Wang J et al (2024) Diabetes mellitus: association of cystatin C- versus creatinine-based estimated glomerular filtration rate with mortality and cardiovascular events. Nephrol Dial Transplant 39(8):1322–1332. 10.1093/ndt/gfae011 38317440 10.1093/ndt/gfae011

[10] Cheuiche AV, Queiroz M, Azeredo-da-Silva ALF, Silveiro SP (2019) Performance of cystatin c-based equations for estimation of glomerular filtration rate in diabetes patients: a PRISMA-compliant systematic review and meta-analysis. Sci Rep 9(1):1418. 10.1038/s41598-018-38286-9 30723243 10.1038/s41598-018-38286-9 PMC6363744

[11] Laucyte-Cibulskiene A, Nilsson PM, Engstrom G, Christensson A (2022) Increased fat mass index is associated with decreased glomerular filtration rate estimated from cystatin C. Data from Malmo Diet and Cancer cohort. PLoS One 17(7):e0271638. 10.1371/journal.pone.0271638 35862349 10.1371/journal.pone.0271638 PMC9302820

[12] Davis TM, Bruce DG, Davis WA (2013) Cohort profile: the Fremantle Diabetes Study. IntJ Epidemiol 42(2):412–421. 10.1093/ije/dys065 22544845 10.1093/ije/dys065

[13] Davis WA, Peters KE, Makepeace A et al (2018) Prevalence of diabetes in Australia: insights from the Fremantle Diabetes Study Phase II. Intern Med J 48(7):803–809. 10.1111/imj.13792 29512259 10.1111/imj.13792 PMC6037554

[14] Krakauer NY, Krakauer JC (2018) Anthropometrics, metabolic syndrome, and mortality hazard. J Obes 2018:9241904. 10.1155/2018/9241904 30123583 10.1155/2018/9241904 PMC6079473

[15] Levey AS, Stevens LA, Schmid CH et al (2009) A new equation to estimate glomerular filtration rate. Ann Int Med 150(9):604–612 19414839 10.7326/0003-4819-150-9-200905050-00006 PMC2763564

[16] Inker LA, Schmid CH, Tighiouart H et al (2012) Estimating glomerular filtration rate from serum creatinine and cystatin C. N Engl J Med 367(1):20–29. 10.1056/NEJMoa1114248 22762315 10.1056/NEJMoa1114248 PMC4398023

[17] Holman CDAJ, Bass AJ, Rosman D et al (2008) A decade of data linkage in Western Australia: strategic design, applications and benefits of the WA data linkage system. Aust Health Rev 32(4):766–777 18980573 10.1071/ah080766

[18] Charlson M, Szatrowski TP, Peterson J, Gold J (1994) Validation of a combined comorbidity index. J Clin Epidemiol 47(11):1245–1251. 10.1016/0895-4356(94)90129-5 7722560 10.1016/0895-4356(94)90129-5

[19] Sudlow C, Gallacher J, Allen N et al (2015) UK Biobank: an open access resource for identifying the causes of a wide range of complex diseases of middle and old age. PLoS Med 12(3):e1001779. 10.1371/journal.pmed.1001779 25826379 10.1371/journal.pmed.1001779 PMC4380465

[20] Carstensen B, Ronn PF, Jorgensen ME (2020) Prevalence, incidence and mortality of type 1 and type 2 diabetes in Denmark 1996–2016. BMJ Open Diabetes Res Care 8(1):e001071. 10.1136/bmjdrc-2019-001071 32475839 10.1136/bmjdrc-2019-001071 PMC7265004

[21] Rajapaksa R, Davis WA, Davis TME (2023) Comparative mortality and its determinants in community-based people with type 1 diabetes: the Fremantle Diabetes Study Phase I. BMJ Open Diabetes Res Care 11(4):e003501. 10.1136/bmjdrc-2023-003501 37487648 10.1136/bmjdrc-2023-003501 PMC10373675

[22] Hwang JA, Song Y, Shin J et al (2022) Changes in mortality according to creatinine/cystatin C ratio in chronic kidney disease and non-chronic kidney disease patients. Front Med (Lausanne) 9:810901. 10.3389/fmed.2022.810901 35308546 10.3389/fmed.2022.810901 PMC8924519

[23] Oberg CM, Lindstrom M, Grubb A, Christensson A (2021) Potential relationship between eGFR(cystatin C) /eGFR(creatinine) -ratio and glomerular basement membrane thickness in diabetic kidney disease. Physiol Rep 9(13):e14939. 10.14814/phy2.14939 34254743 10.14814/phy2.14939 PMC8276256

[24] Ide H, Iwase M, Fujii H et al (2017) Comparison of cystatin C- and creatinine-based estimated glomerular filtration rates for predicting all-cause mortality in Japanese patients with type 2 diabetes: the Fukuoka Diabetes Registry. Clin Exp Nephrol 21(3):383–390. 10.1007/s10157-016-1296-2 27339449 10.1007/s10157-016-1296-2

[25] Levin A, Stevens PE (2014) Summary of KDIGO 2012 CKD Guideline: behind the scenes, need for guidance, and a framework for moving forward. Kidney Int 85(1):49–61. 10.1038/ki.2013.444 24284513 10.1038/ki.2013.444

[26] Levin A, Ahmed SB, Carrero JJ et al (2024) Executive summary of the KDIGO 2024 Clinical Practice Guideline for the Evaluation and Management of Chronic Kidney Disease: known knowns and known unknowns. Kidney Int 105(4):684–701. 10.1016/j.kint.2023.10.016 38519239 10.1016/j.kint.2023.10.016

[27] Luis-Lima S, Higueras Linares T, Henriquez-Gomez L et al (2019) The error of estimated GFR in type 2 diabetes mellitus. J Clin Med 8(10):1543. 10.3390/jcm8101543 31561432 10.3390/jcm8101543 PMC6832380

[28] National Institute for Health and Care Excellence (2021) Chronic kidney disease: assessment and management. Available from https://www.nice.org.uk/guidance/ng203. Accessed 1 June 2024 34672500

